# Circular RNA *hsa_circ_0008726* Targets the *hsa-miR-206-3p*/KLF4 Axis to Modulate 4,4′-Methylene Diphenyl Diisocyanate-Glutathione Conjugate-Induced Chemokine Transcription in Macrophages

**DOI:** 10.3390/cells13201725

**Published:** 2024-10-18

**Authors:** Chen-Chung Lin, Brandon F. Law, Justin M. Hettick

**Affiliations:** Allergy and Clinical Immunology Branch, Health Effects Laboratory Division, National Institute for Occupational Safety and Health, Morgantown, WV 26505, USA; bhl7@cdc.gov (B.F.L.); ayf2@cdc.gov (J.M.H.)

**Keywords:** 4,4′-methylene diphenyl diisocyanate (MDI), circular RNAs (circRNAs), *hsa-miR-206-3p*, occupational asthma (OA), Krüppel-like factor 4 (KLF4)

## Abstract

Exposure to 4,4′-methylene diphenyl diisocyanate (MDI) in the workplace may lead to the development of occupational asthma (OA). However, the specific mechanism(s) by which MDI induces OA are poorly understood. Previous reports have demonstrated that MDI and MDI-glutathione (GSH) conjugate exposure downregulates endogenous human/murine (*hsa/mmu*)-microRNA*(miR)-206-3p*, resulting in the activation of *mmu/hsa-miR-206-3p*-regulated signaling pathways in macrophages. Circular RNAs (circRNAs) regulate many important biological processes by targeting endogenous miRs; however, whether MDI/MDI-GSH exposure may influence circRNA expressions is unknown. Several circRNAs have been identified that regulate *hsa-miR-206-3p*. We hypothesize that MDI-GSH conjugate exposure induces endogenous circRNA(s) to regulate *hsa-miR-206-3p* in macrophages. The expression of candidate *hsa-miR-206-3p*-binding circRNAs was determined from MDI-GSH conjugate-treated differentiated THP-1 macrophages using RT-qPCR. MDI-GSH exposures induced *hsa_circ_0008726* and its host gene transcript *DNAJB6*, whereas other circRNA(s) examined were either not detected or unchanged. RNA-induced silencing complex-immunoprecipitation (RISC-IP) experiments confirm that *hsa-miR-206-3p* can bind to *hsa_circ_0008726*. The expressions of endogenous *hsa-miR-206-3p*, *hsa-miR-206-3p*-regulated *KLF4*, and KLF4-activated M2 macrophage-associated markers and chemokines were up-/down-regulated by transfection of *hsa_circ_0008726* siRNAs or *hsa_circ_0008726* overexpression plasmid in macrophages, respectively. These results suggest MDI-GSH exposure downregulates *hsa-miR-206-3p* via induction of endogenous *hsa_circ_0008726/DNAJB6*, resulting in the upregulation of *hsa-miR-206-3p*-mediated regulations in macrophages.

## 1. Introduction

Diisocyanates are highly reactive crosslinking agents utilized in the manufacture of polyurethane products [1]. Of these, 4,4′-methylene diphenyl diisocyanate (MDI) is the most widely utilized, and global demand for MDI is predicted to continue to grow [2]. Workplace exposure to MDI is a leading cause of occupational asthma (OA) development [3,4,5,6,7,8]. However, the specific molecular mechanism(s) through which MDI exposure causes OA remains an active area of research.

In the lung microenvironment, the airway fluid contains a very high concentration of the antioxidant glutathione (GSH) (>100 µM) [9], and evidence suggests that the free thiol groups of GSH are a primary target of isocyanate *in vivo* [10,11,12]. Isocyanate reacts with the free thiol of GSH to a thiocarbamate; further reaction with water may reverse the thiocarbamate linkage and hydrolyze the original isocyanate moiety to an amine group [11]. Thiocarbamate species may further react with endogenous proteins via transcarbamoylation that may contribute to the isocyanate-mediated irritation/allergy reaction of the cells within the lung microenvironment.

Following allergen exposure, airway immune cells infiltrate into the lung microenvironment, and interactions with airway cells are important for asthma pathogenesis [13,14,15]. Among the first immune cells to respond to inhaled allergens are the alveolar macrophages, which have been implicated in the development of asthma [16]. Alternatively activated (M2) macrophage populations have been shown to be elevated in the airways of asthmatic patients [17]. *In vivo*/*vitro* studies have indicated that MDI/MDI-GSH conjugate exposure led to the induction of M2 macrophage-associated gene signatures [18,19], and some of the M2 macrophage-associated genes are induced partially through Krüppel-like factor 4 (KLF4)-mediated transcriptional activation [20]. Macrophage differentiation and M2 macrophage polarization can be regulated by KLF4 [21]; however, the detailed molecular mechanism(s) by which MDI/MDI-GSH conjugate exposure induces KLF4 and KLF4-mediated M2 macrophage-associated gene activation remains an active research area. Prior studies demonstrate that endogenous microRNAs (miRs) may play a role in regulation of the MDI/MDI-GSH conjugate-mediated induction of KLF4 in macrophages [22]. However, the molecular mechanism through which MDI/MDI-GSH conjugate exposure alters miR levels in macrophages is currently unknown.

Circular RNAs (circRNAs) are noncoding RNAs with a circular structure that have been suggested to participate in the pathogenesis of many different diseases, such as prostate cancer, arthritis, diabetes, and asthma [23,24,25,26,27], through competing endogenous RNA (ceRNA) mechanisms [28], including acting as a miR sponge to regulate gene expression. Moreover, endogenous circRNA species have been reported to regulate macrophage polarization and are involved in the pathogenesis of many different diseases. Endogenous circRNAs are differentially expressed and can regulate macrophage polarization by targeting miR-mediated macrophage polarization-associated gene regulation [29]. For example, circRNA *hsa_circ_0005567* promotes M2 macrophage polarization by sponging *hsa-miR-492* and inducing suppressor of cytokine signaling 2 (SOCS2) expression in osteoarthritis [30]. Inhibition of circRNA *hsa_circ_0074854* suppressed exosome-mediated macrophage M2 polarization in liver cancer, indicating that *hsa_circ_0074854* promotes macrophages towards the M2 phenotype [31]. Additionally, circRNAs have been shown to participate in classically activated (M1) macrophage polarization, as the circRNA *circPPM1F* promotes M1 macrophage polarization in type 1 diabetes mellitus [32]. Furthermore, circular RNA *circCdyl* promotes M1 macrophage polarization by sponging *miR-let-7c* in aortic aneurysm [33]. However, the roles of circRNAs in MDI and MDI-GSH conjugate-mediated induction of M2 macrophage-associated gene expression have not been reported.

Previously, we determined that MDI-GSH conjugate exposure leads to downregulation of two endogenous miRs, *mmu/hsa-miR-206-3p* and *mmu/hsa-miR-381-3p* [34,35], and upregulation of endogenous KLF4 and KLF4-mediated M2 macrophage-associate genes in human macrophages [20]. However, the specific mechanism by which MDI downregulates endogenous *hsa-miR-206-3p* and *hsa-miR-381-3p* is currently unknown. One of the many functions of endogenous circRNAs is degradation/downregulation of endogenous miRs through binding [36]. Several circRNAs have been shown to regulate endogenous *hsa-miR-206-3p* levels through ceRNA mechanisms and *hsa-miR-206-3p*-mediated downstream gene regulation, including *hsa_circ_0000199* [37], *hsa_circ_0001264* [38], *hsa_circ_0001982* [39], *hsa_circ_0004662* [40], *hsa_circ_0007428* [41], *hsa_circ_0008726* [42,43], *hsa_circ_0056618* [44,45], *hsa_circ_0057558* [24], *hsa_circ_0058141* [46], and *hsa_circ_0072088* [47]. Therefore, we hypothesize that MDI (in the form of MDI-GSH conjugate) exposure downregulates endogenous *hsa-miR-206-3p* via induction of certain endogenous circRNA(s) in macrophages.

This report focuses on characterizing MDI in the form of MDI-GSH conjugate-mediated candidate *hsa-miR-206-3p* binding circRNA(s) responses and verifying the interaction between *hsa-miR-206-3p* and candidate circRNA(s). Furthermore, we characterize the downstream molecular mechanistic effects of MDI-mediated circRNA changes that contribute to the regulation of KLF4 and KLF4-mediated regulation of downstream M2 macrophage-associated gene responses. *In vitro* exposure to MDI-GSH conjugates results in downregulation of endogenous *hsa-miR-206-3p*, upregulation of *hsa_circ_0008726* and *hsa_circ_0008726* host RNA transcript, DnaJ Heat Shock Protein Family (Hsp40) Member B6 (*DNAJB6*), with subsequent upregulation of KLF4-mediated induction of M2 macrophage-associated genes. Here, we provide a putative circRNA-regulated mechanism to describe downregulation of endogenous *hsa-miR-206-3p* after MDI-GSH conjugate exposure in macrophages.

## 2. Materials and Methods

### 2.1. Caution

The 4,4′-methylene diphenyl diisocyanate (MDI) is a strongly reactive, hazardous, irritating, and well-known immune sensitizing chemical. Appropriate personal protective equipment (PPE) such as nitrile gloves, protective clothing, and goggles must be utilized while handling MDI.

### 2.2. Chemicals and Reagents

HPLC grade acetone (CAS No. 67-64-1), 4,4′-methylene diphenyl diisocyanate (MDI, 98%) (CAS No. 101-68-8), 3Å molecular sieve (4–8 mesh), Tween-20 (CAS No. 9005-64-5), dimethyl sulfoxide (DMSO) (CAS No. 67-68-5), phorbol 12-myristate 13-acetate (PMA) (CAS No. 16561-29-8), butyric acid (Cas No. 107-92-6), bovine serum albumin (BSA), tris-buffered saline (TBS), and reduced-glutathione (GSH) (CAS No. 70-18-8) were acquired from MilliporeSigma (St. Louis, MO, USA). Roswell Park Memorial Institute (RPMI)-1640 culture medium, phosphate-buffered saline (PBS), and penicillin–streptomycin–glutamine (PSG; 100×) were acquired from ThermoFisher Scientific (Waltham, MA, USA). Hyclone^™^ fetal bovine serum (FBS) was obtained from Cytiva Life Sciences (Marlborough, MA, USA). Dry acetone was prepared by incubating HPLC-grade acetone with 3Å molecular sieves for a minimum of 24 h to adsorb water.

### 2.3. Cell Culture and Differentiation

Cell culture and differentiation were performed as previously described [48]. THP-1 (ATCC^®^ TIB-202^™^), Clone 15 HL-60 (HL-60_C15; ATCC^®^ CRL-1964^™^), and Jurkat Clone E6-1 (Jurkat_E6-1; ATCC^®^ TIB-152^™^) cells were acquired from American Type Culture Collection (ATCC; Manassas, VA, USA) and maintained at 0.5–1 × 10^6^/mL in complete RPMI media (RPMI-1640 media supplement, 10% FBS, 1 × PSG) at 37 °C in a humidified atmosphere with 5% CO_2_ [48]. THP-1 cells (2 × 10^6^ cells) were differentiated using 10 ng/mL PMA in 10 cm culture dishes for 72 h. The PMA-differentiated THP-1 macrophages were enhanced by washing twice with PBS following removal of PMA and incubation of the cells in PMA-free fresh complete media for another 72 h. Differentiation by PMA at a concentration of 10 ng/mL has been shown to enhance response to polarizing stimuli [49,50]. All *in vitro* cell experiments described in this study used differentiated/enhanced THP-1 macrophages. For eosinophil differentiation in chemotaxis experiments, HL-60_C15 cells (5 × 10^5^ cells/mL) were cultured in complete RPMI-1640 media containing 0.5 mM butyric acid for 7 days as previously described [51,52,53].

### 2.4. MDI-GSH Conjugation Reactions

MDI-GSH conjugate was prepared as previously described [34,48]. Briefly, 10 mM GSH in 200 mM sodium phosphate buffer (pH = 7.4) was prepared. Dry acetone was freshly prepared, and 50 µL of 10% MDI (*w*/*v*) in dry acetone were added to 25 mL of GSH solution dropwise with stirring, resulting in an approximate MDI concentration of 800 µM. MDI-GSH conjugates reactions were performed at 25 °C with end-over-end mixing for 1 h and then centrifuged for 5 min at 10,000× *g* and filtered with a 0.2 µm syringe filter. Freshly prepared MDI-GSH conjugate was immediately added into differentiated/enhanced THP-1 macrophages at 0, 1, and 10 µM as indicated or at 10 µM MDI concentrations.

### 2.5. Plasmid Construction

Plasmid construction was performed similar to our previous reports [22,34]. The circular RNA overexpression vector pcDNA3.1^(+)^_CircRNA_Mini Vector was obtained from Addgene (plasmid id#60648; Watertown, MA, USA), which was deposited by Dr. Jeremy Wilusz at Baylor College of Medicine [54]. To generate a wildtype (WT) *hsa_circ_0008726* overexpression plasmid, a 0.28 kb cDNA fragment representing the full length of human circular RNA *hsa_circ_0008726* was generated by PCR using a *PacI* restriction site containing forward primer (cccttaattaaATATCGGAAACTGGCACTG) and a *SacII* restriction site containing reverse primer (cccccgcggCCCATTTTCATTTGACTTC) on THP-1 cell cDNA. The PCR amplified *hsa_circ_0008726* cDNA fragment was treated with *PacI* and *SacII*. This fragment was inserted into pcDNA3.1^(+)^_CircRNA_Mini Vector that was prepared by sequential enzyme treatments with *PacI*, *SacII*, and calf intestinal alkaline phosphatase (CIP) to generate pcDNA3.1^(+)^_Circ_Mini-*hsa_circ_0008726* overexpression plasmid.

### 2.6. Plasmid, microRNA Mimics/Inhibitors, and siRNA Transfection

Nucleic acid transfections were performed as previously described [20,34,48]. Briefly, for the circRNA overexpression study, 1 × 10^6^ differentiated/enhanced THP-1 macrophages were reverse transfected with 2.5 µg of either pcDNA3.1^(+)^_CircRNA_Mini-*hsa_circ_0008726* or pcDNA3.1^(+)^_CircRNA_Mini Vector using Mirus *Trans*IT^®^-2020 transfection reagent for 48 h, after which total RNA was isolated using the *mirVana*^™^ miR Isolation Kit (ThermoFisher Scientific, Waltham, MA, USA) according to the manufacturer’s instructions. For functional analyses on binding and regulation with *hsa_circ_0008726*, the following *mirVana*^™^ miRNA inhibitors (MH; Cat#4464084) and miR-mimics (MC; Cat#4464066) were obtained from ThermoFisher Scientific and diluted to 20 µM in nuclease-free water: *hsa-miR-206-3p* (MH10409, MC10409), *hsa-miR-381-3p* (MH10242, MC10242), *mirVana*^™^ miRNA Inhibitor, negative control #1 (4464076), and *mirVana*^™^ miRNA Mimic, negative control #1 (4464058). For circRNA *hsa_circ_0008726* knockdown studies, two Dharmacon custom siRNAs to *hsa_circ_0008726* and ON-TARGETplus Non-targeting Control Pool (Cat# D-001810-10-05) were purchased from Horizon Discovery (Lafayette, CO, USA). The custom-designed siRNA sequences are as follows: si-*hsa_circ_0008726*#1 (Sense): 5′-ACUUCUUUGAUAUCGGAAACUUU-3′; si-*hsa_circ_0008726*#1 (Antisense) 5′-AGUUUCCGAUAUCAAAGAAGUUU-3′; si-*hsa_circ_0008726*#2 (Sense): 5′-UUUGACUUCUUUGAUAUCGGAUU-3′; si-*hsa_circ_0008726*#2 (Antisense): 5′-UCCGAUAUCAAAGAAGUCAAAUU-3′. As previously described, differentiated/enhanced THP-1 macrophages were reverse transfected and forward transfected after 24 h [55]. Total RNA was prepared for RT-qPCR expression analyses 24 h following forward transfection.

### 2.7. Expression Analyses

To determine whether endogenous circRNA *hsa_circ_0008726* is expressed within differentiated/enhanced THP-1 macrophages, total RNA was extracted from THP-1 macrophages using the *mirVana*^™^ miR Isolation Kit (ThermoFisher Scientific). To deplete linear RNA species in isolated THP-1 total RNA, 3 µg of purified THP-1 total RNA was treated with 20 U Ribonuclease R (RNase R; LGC Biosearch Technologies, Teddington, UK). Briefly, a 20 µL reaction mix containing 3 µg of total RNA, 20 U of RNase inhibitor (Cat# N8080119; ThermoFisher Scientific), 1 × RNase R buffer, and 20 U of RNase R was incubated for 30 min at 37 °C. Control reactions were performed similarly, but without RNase R. After RNAase R treatment, the processed RNA was further purified by using the *mirVana*^™^ miR Isolation Kit according to the manufacturer’s instructions. RNase R protein was removed during the RNA isolation step using the *mirVana*^™^ miR Isolation Kit, which eliminates all protein from the sample. Total RNA with or without RNAse R treatment was reverse transcribed to cDNA using a High-Capacity cDNA Synthesis Kit (Thermo Fisher Scientific) according to the manufacturer’s instructions. PCR reactions were conducted using either *DNAJB6* convergent primers (*DNAJB6*-F: TAAAGTCCTTAACAATAAATG; *DNAJB6*-R: GAGGCCGGCAGGCTGGGCTGGC) or circRNA *hsa_circ_0008726* divergent primers (see Supplemental Table S1).

The mRNA, circRNA, and miR levels from THP-1 total RNA were analyzed using RT-qPCR like those previously described [20,22,34,35,48,55]. Briefly, all RT-qPCR reactions were normalized to human beta-2 microglobulin (*B2M*) for mRNA analysis or to *U6* snRNA for circRNA and miR analysis. Gene expression and miR-specific assays were purchased from ThermoFisher Scientific and include human *KLF4* (Cat#4331182; Assay ID: Hs00358836_m1), *DNAJB6* (Hs00369717_m1), *CD206* (Hs00267207_m1), *TGM2* (Hs01096681_m1), *CCL17* (Hs00171074_m1), *CCL22* (Hs01574247_m1), *CCL24* (Hs00171082_m1), *B2M* (Hs00187842_m1), *hsa-miR-206-3p* (Cat# 4427975; Assay ID No. 000510; *hsa*/*Homo sapiens*), and *U6* snRNA (No. 001973). For circRNA assays, SYBR Green-based qPCR reactions were performed using PowerTrack^™^ SYBR Green Master Mix from ThermoFisher Scientific (Cat#A46110) with divergent primer sets listed in Supplemental Table S1 and normalized using *U6* snRNA with the following primer sequences: *U6_*snRNA-F: CTCGCTTCGGCAGCACA; *U6_*snRNA-R: AACGCTTCACGAATTTGCGT. PCR reactions were performed using an ABI 7500 Real-Time PCR System (ThermoFisher Scientific). Expressions of mRNAs and miRs were determined using the ^ΔΔ^CT method [34].

### 2.8. Validation of miR-circRNA Interaction by Argonaute (AGO) Immunoprecipitation

The miR-containing RNA-inducing silencing complex (miR/RISC) and miR-targeting circRNA(s) were immunoprecipitated using the miRNA target IP kit (Active Motif, Carlsbad, CA, USA) as previously described [20,34,48]. Briefly, differentiated/enhanced THP-1 macrophages were trypsinized and seeded at 8 × 10^6^ cells in a 10 cm dish. Macrophages were transfected with 25 nM of either miR-mimic-206-3p, miR-mimic-381-3p, or miR-mimic negative control #1 for 24 h. Two 10 cm dishes using an equal number of 1.6 × 10^7^ cells were utilized for each IP reaction. Cells were lysed, and the lysates were divided into two equal aliquots. Aliquots underwent IP using either a pan-AGO antibody (to precipitate the RISC containing AGOs/miRs/mRNAs/circRNAs) or an isotype IgG antibody control. The precipitate was collected, and RNA was purified from the RISC complex using the *mirVana*^™^ miR Isolation Kit (ThermoFisher Scientific). RNA was reverse transcribed to cDNA using the High-Capacity cDNA Reverse Transcription Kit (ThermoFisher Scientific), and *hsa_circ_0008726* divergent primer was mixed with PowerTrack^™^ SYBR Green Master Mix for RT-qPCR reactions. Data were analyzed by comparing the cells transfected with miR-mimics or non-target miR-mimic-control, and the fold enrichment of *hsa_circ_0008726* was calculated from the anti-panAGO and the IgG isotype antibody IP preparations as per the manufacturer’s instructions.

### 2.9. Chemokine ELISA

Secreted CCL17, CCL22, and CCL24 protein concentrations in conditioned media were determined as previously described [20]. Briefly, conditioned media was collected 48 h following THP-1 macrophage transfection with 2.5 µg of either pcDNA3.1^(+)^_CircRNA_Mini-*hsa_circ_0008726* overexpression plasmid or empty pcDNA3.1^(+)^_CircRNA_Mini vector. The following enzyme-linked immunosorbent assay (ELISA) kits were obtained from R&D systems (Minneapolis, MN, USA): human CCL17/TARC ELISA kit (Cat#DY364); human CCL22/MDC (#DY336); and human CCL24/Eotaxin-2 (#DY343). The sensitivity for each chemokine is as follows: CCL17 (7.8 pg/mL), CCL22 (7.8 pg/mL), and CCL24 (31.2 pg/mL). Human CCL17, CCL22, and CCL24 chemokines released into the conditioned media from plasmid-transfected THP-1 macrophages were determined by ELISA per manufacturer’s directions.

### 2.10. Chemotaxis Assays and Quantification of Migrated Cells

Chemotaxis/cell migration in response to conditioned media collected from THP-1 macrophages treated with pcDNA3.1^(+)^_CircRNA_Mini-*hsa_circ_0008726* overexpression plasmid or pcDNA3.1^(+)^_CircRNA_Mini vector only were performed as previously described [20,48]. Chemotaxis/cell migration assays were performed on a 24-well plate with Transwell^™^ inserts (3 µm pore, Corning^™^ Transwell^™^ plates, ThermoFisher Scientific). Moreover, 1 × 10^6^ naive T-cells (Jurkat_E6-1 T cells) or eosinophils (butyric acid-differentiated HL-60_C15 cells) in 100 µL serum-free RPMI 1640 media were added to the upper chamber. Moreover, 500 µL of cell-free conditioned media from either pcDNA3.1^(+)^_CircRNA_Mini-*hsa_circ_0008726* overexpression plasmid or pcDNA3.1^(+)^_CircRNA_Mini vector plasmid transfected THP-1 macrophages were placed in the lower chamber as a chemoattractant. Immune cells were incubated for 6 h at 37 °C in a humidified atmosphere with 5% CO_2_. The cells that migrated into the lower chamber media were collected and stored on ice. Cells that failed to migrate from the upper chamber were aspirated and discarded. The Transwell assembly was washed twice with PBS. Furthermore, 500 µL of cell detaching media (0.25% Trypsin-EDTA, Cat#25200056, ThermoFisher Scientific) were added back to the lower chambers with the upper chambers reinstalled. The whole plate was further incubated at 37 °C for 30 min to detach any remaining migrated cells. After 30 min, the detached cells were combined with the conditioned media/migrated cells previously collected, centrifuged at 300× *g* for 5 min, washed with PBS twice, and stored at −80 °C. Total migrated cell numbers in the lower chamber were quantified by using the CyQUANT^®^ Cell proliferation assay (ThermoFisher Scientific) according to the manufacturer’s directions.

### 2.11. Statistical Analysis

Statistical analysis was performed as previously described [20,34,48]. Briefly, data were analyzed using either the unpaired *t*-test (two-tailed) when comparing two groups or one-way analysis of variance followed by Tukey’s multiple comparison ad hoc post-test when comparing multiple groups. Statistical analyses were performed in GraphPad Prism 7.0 (GraphPad Software, La Jolla, CA, USA). *p* < 0.05 was used to assign significance.

## 3. Results

### 3.1. MDI-GSH Conjugate Exposure Upregulates Endogenous Circular RNA hsa_circ_0008726 in Differentiated/Enhanced THP-1 Human Macrophages

**Figure 1:**
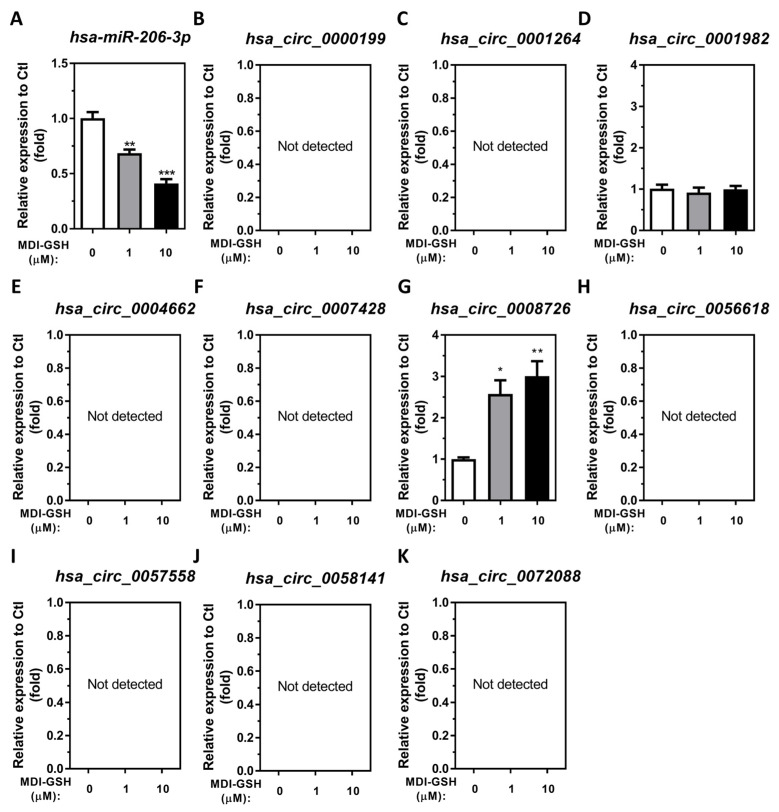
**MDI-GSH conjugate treatment induces endogenous circRNA *hsa_circ_0008726* in differentiated/enhanced THP-1 macrophages.** Total RNA was isolated from the indicated MDI-GSH conjugate-treated differentiated/enhanced THP-1 macrophages by the *mirVana*^™^ miR isolation kit, reverse transcribed, and subjected to SYBR green-based or TaqMan stem-loop miR RT-qPCR. Endogenous miR/circRNA expressions of (**A**) *hsa-miR-206-3p*, (**B**) *hsa_circ_0000199*, (**C**) *hsa_circ_0001264*, (**D**) *hsa_circ_0001982*, (**E**) *hsa_circ_0004662*, (**F**) *hsa_circ_0007428*, (**G**) *hsa_circ_0008726*, (**H**) *hsa_circ_0056618*, (**I**) *hsa_circ_0057558*, (**J**) *hsa_circ_0058141*, and (**K**) *hsa_circ_0072088* were determined 24 h after MDI-GSH conjugate treatments (N = 3; bars, SEM). MDI: 4,4′-methylene diphenyl diisocyanate. GSH: Glutathione. (* *p* < 0.05, ** *p* < 0.01, *** *p* < 0.001).

Previous reports from our laboratory have shown that *in vivo*/*in vitro* MDI/MDI-GSH conjugate exposure downregulates endogenous *mmu/hsa-miR-206-3p* in bronchoalveolar lavage cells (BALCs) isolated from an MDI-exposed mouse model and in an acute MDI-GSH conjugate exposed THP-1 macrophage cell culture model [34,48]. These results indicate that MDI and MDI-GSH conjugates cause endogenous *mmu/hsa-miR-206-3p* downregulation via an unknown mechanism in alveolar macrophages. Through a literature search (10/2023), we identified several endogenous circRNA candidates that have been reported to regulate endogenous *hsa-miR-206-3p* levels and function in diverse cell types. The candidate *hsa-miR-206-3p* targeting circRNAs include *hsa_circ_0000199* [37], *hsa_circ_0001264* [38], *hsa_circ_0001982* [39], *hsa_circ_0004662* [40], *hsa_circ_0007428* [41], *hsa_circ_0008726* [42,43], *hsa_circ_0056618* [44,45], *hsa_circ_0057558* [24], *hsa_circ_0058141* [46], and *hsa_circ_0072088* [47]. To investigate whether MDI-GSH conjugate exposure can cause expression changes in candidate *hsa-miR-206-3p* sponging/targeting circRNAs, we utilized differentiated/enhanced human THP-1 macrophages in an *in vitro* cell model to investigate the circRNA responses to MDI-GSH conjugate exposure and downstream cellular signaling pathways. Differentiated/enhanced THP-1 macrophages were treated with MDI-GSH conjugate (0, 1, and 10 µM) for 24 h. The endogenous *hsa-miR-206-3p* and candidate circRNA levels were measured by RT-qPCR using either the TaqMan *hsa-miR-206-3p* assay or designed circRNA primers (Supplemental Table S1). In agreement with our previous findings, endogenous *hsa-miR-206-3p* levels are significantly downregulated following MDI-GSH conjugate exposure from 1.46-fold to 2.43-fold, respectively (Figure 1A). Of the 10 candidate *hsa-miR-206-3p* sponging/targeting circRNAs identified from the literature, MDI-GSH conjugate treatment significantly upregulated endogenous *hsa_circ_0008726* levels by 2.57- to 3.01-fold (Figure 1G), whereas the endogenous *hsa_circ_0001982* levels remained unchanged (Figure 1D). We failed to detect other reported candidate *hsa-miR-206-3p* sponging/targeting circRNAs *hsa_circ_0000199*, *hsa_circ_0001264*, *hsa_circ_0004662*, *hsa_circ_0007428*, *hsa_circ_0056618*, *hsa_circ_0057558*, *hsa_circ_0058141*, and *hsa_circ_0072088* in differentiated/enhanced THP-1 macrophages exposed to MDI-GSH conjugate (Figure 1B,C,E,F,H–K). These results suggest that exposure to MDI-GSH conjugate results in upregulation of endogenous *hsa_circ_0008726* and downregulation of *hsa-miR-206-3p* in macrophages.

### 3.2. MDI-GSH Conjugates Upregulate the Endogenous hsa_circ_0008726 Host Gene DNAJB6 RNA Transcript in Macrophages

**Figure 2:**
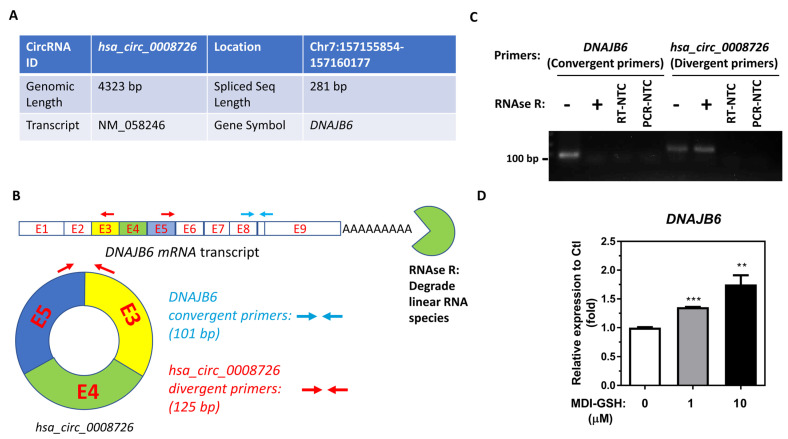
**Circular RNA *hsa_circ_0008726* is presented in THP-1 macrophages, and MDI-GSH conjugates upregulate endogenous *hsa_circ_0008726* parental host gene transcript *DNAJB6*.** (**A**) Characteristics of *hsa_circ_0008726* obtained from the Circular RNA Interactome database. (**B**) Illustration shows exon numbers and designed convergent and divergent primer sites on the mature *DNAJB6* transcripts. RNAse R degrades linear RNA species, including the *DNAJB6* transcript. CircRNA *hsa_circ_0008726* is back spliced from exon 3–5 of the *DNAJB6* transcript. (**C**) Total RNA was isolated from THP-1 macrophages by the *mirVana^™^* miR isolation kit and treated with or without RNAse R, further purified using the *mirVana^™^* miR isolation kit, reverse transcribed, and subjected to RT-PCR using convergent or divergent primers. RT-NTC: Templates from a cDNA synthesis reaction without adding reverse transcriptase. PCR-NTC: Use only water to replace cDNA templates during PCR reaction. (**D**) Total RNA was isolated from MDI-GSH-treated differentiated/enhanced THP-1 macrophages at indicated concentrations for 24 h by the *mirVana^™^* miR isolation kit, reverse transcribed, and subjected to TaqMan RT-qPCR assays. Endogenous levels of *DNAJB6* were determined at 24 h after MDI-GSH conjugate treatment (N = 3; bars, SEM). MDI: 4,4′-methylene diphenyl diisocyanate. GSH: Glutathione (** *p* < 0.01, *** *p* < 0.001).

*In silico* query of the Circular RNA Interactome database (https://circinteractome.nia.nih.gov/) [56] reveals that mature *hsa_circ_0008726* consists of 281 nucleotides and is backspliced from the host gene *DNAJB6* located at chromosome 7 (Figure 2A). The mature *DNAJB6* mRNA transcript (NM_058246) consists of nine different exons after splicing. Mature *hsa_circ_0008726* is derived from exons 4, 5, and 6 of the *DNAJB6* pre-mRNA transcript via a backsplicing mechanism during *DNAJB6* mRNA maturation in the nucleus (Figure 2B). To determine whether or not *hsa_circ_0008726* is indeed expressed in differentiated/enhanced THP-1 macrophages, we treated total RNA isolated from differentiated/enhanced THP-1 macrophages with RNAse R to deplete the linear RNA species and performed RT-PCR using *DNAJB6* convergent primers (to detect mature *DNAJB6* mRNA) and the *hsa_circ_0008726* divergent primers (to detect the mature form of *hsa_circ_0008726* including the backsplice junction) (Figure 2B). We were able to detect *hsa_circ_0008726* in both THP-1 RNAs treated with and without RNAse R using *hsa_circ_0008726* divergent primers; however, we did not detect *hsa_circ_0008726* host *DNAJB6* mRNA expression in RNAse R-treated THP-1 RNA using the *DNAJB6* convergent primers (Figure 2C). The PCR results indicate that *hsa_circ_0008726* is indeed expressed in differentiated/enhanced THP-1 macrophages. To determine whether MDI-GSH conjugate exposure results in upregulation of *hsa_circ_0008726* via induction of *DNAJB6* RNA transcript, differentiated/enhanced THP-1 macrophages were treated with 0, 1, and 10 µM MDI-GSH conjugate for 24 h. The endogenous *DNAJB6* transcripts were significantly upregulated from 1.36- to 1.75-fold (Figure 2D), indicating that *hsa_circ_0008726* is expressed in THP-1 macrophages and MDI-GSH conjugate exposure may upregulate endogenous *hsa_circ_0008726* through induction of *hsa_circ_0008726* host *DNAJB6* RNA transcript in macrophages.

### 3.3. Circular RNA hsa_circ_0008726 Interacts with hsa-miR-206-3p in THP-1 Macrophages

**Figure 3:**
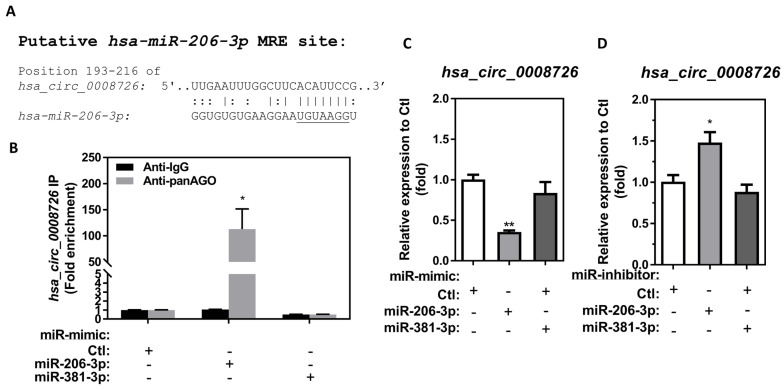
**Human circular RNA *hsa_circ_0008726* is a target of *hsa-miR-206-3p.*** (**A**) Alignment of the *hsa_circ_0008726* sequence regions of potential *hsa-miR-206-3p* binding sites. (**B**) Differentiated/enhanced THP-1 macrophages were transfected with 25 nM of indicated miR-mimic or nontargeting miR-mimic control (miR-mimic-Ctl) for 24 h. The cells were collected and immunoprecipitated using the panAGO or isotype IgG antibody after 24 h transfection. RNA was isolated, and the fold enrichment of *hsa_circ_0008726* was measured (N = 3; bars, SEM). (**C**,**D**) THP-1 macrophages were transfected with 25 nM of either miR-mimic/inhibitor-206-3p, miR-mimic/inhibitor-381-3p, or nontargeting miR-mimic/inhibitor control for 24 h. Total RNA was isolated from the indicated miR-mimics/inhibitors transfected THP-1 macrophages by the *mirVana^™^* miR isolation kit, reverse transcribed, and subjected to RT-qPCR. The endogenous *hsa_circ_0008726* levels from indicated (**C**) miR-mimics or (**D**) miR-inhibitors transfected THP-1 macrophages were determined by SYBR Green RT-qPCR assays (N = 3; bars, SEM). (* *p* < 0.05, ** *p* < 0.01).

Previous studies have shown that *hsa_circ_0008726* can target/sponge *hsa-miR-206-3p* in esophageal squamous carcinoma cells and trophoblast cells [42,43]. However, the ability of *hsa_circ_0008726* to regulate *hsa-miR-206-3p* through binding/sponging in macrophages is currently unknown. To further elucidate a potential molecular mechanism through which *hsa_circ_0008726* regulates *hsa-miR-206-3p* via sponging/targeting using microRNA response element (MRE) site(s) on *hsa_circ_0008726*, we examined the potential interaction between *hsa_circ_0008726* and *hsa-miR-206-3p* and *hsa-miR-381-3p* using the *in silico* tool Circbank (http://www.circbank.cn/) [57]. There is one potential *hsa-miR-206-3p* MRE site located on *hsa_circ_0008726* at position 193–216 (Figure 3A); however, no *hsa-miR-381-3p* MRE site was found on *hsa_circ_0008726* (Supplemental Figure S1).

CircRNAs are theoretically stable due to the closed structure that prevents them from degradation by exonucleases, which degrade linear RNAs. Endogenous circRNA degradation can be initiated after the binding of endogenous miRs with the MRE on the circRNA sequences. The binding between miRs and circRNA requires miR-mediated recruitment of the argonaute (AGO) protein containing RNA-induced silencing complexes (RISC) onto the circRNA through the MRE sites. *In vivo*/*vitro* exposure to MDI/MDI-GSH in BALCs and macrophages downregulates endogenous *mmu/hsa-miR-206-3p* and *-381-3p* [34,35]. To determine whether *hsa_circ_0008726* is directly involved in downregulation of either *hsa-miR-206-3p* or *hsa-miR-381-3p*, it was necessary to first confirm the potential *hsa-miR-206-3p* and *hsa-miR-381-3p* binding to circRNA *hsa_circ_0008726* by performing an AGO antibody pulldown to precipitate the RISC containing AGOs/miRs/circRNAs (RISC-IP). This was followed by confirmation of *hsa-miR-206-3p* or *hsa-miR-381-3p* regulation of *hsa_circ_0008726* using gain- and loss-of-function studies by transfecting miR-mimics/inhibitors. Consistent with the *in silico* prediction that *hsa_circ_0008726* contains the MRE site of *hsa-miR-206-3p* but not *hsa-miR-381-3p*, transfection of miR-mimic-206-3p results in increased precipitated *hsa_circ_0008726* by 112.9-fold compared to the non-targeting miR-mimic control. Transfection of miR-mimic-381-3p did not result in increased precipitated *hsa_circ_0008726* transcripts when compared to the non-targeting control (Figure 3B). These results indicate that the *hsa_circ_0008726* transcript binds to RISCs containing *hsa-miR-206-3p* but not *hsa-miR-381-3p*.

To investigate the possibility that *hsa-miR-206-3p* can regulate endogenous *hsa_circ_0008726* through a binding/sponging/miR-mediated degradation mechanism in macrophages, we performed gain- and loss-of-function studies by transfection of either miR-mimics or miR-inhibitors of *hsa-miR-206-3p* and *hsa-miR-381-3p* into differentiated/enhanced THP-1 macrophages. Transfection of miR-mimic-206-3p downregulates endogenous *hsa_circ_0008726* by 2.81-fold (Figure 3C), whereas transfection of miR-inhibitor-206-3p upregulates endogenous *hsa_circ_0008726* by 1.48-fold (Figure 3D) compared to the nontargeting miR-mimic or miR-inhibitor control transfected THP-1 macrophages. To further investigate whether the sponging/binding/miR-mediated degradation mechanism between *hsa-miR-206-3p* and *hsa_circ_0008726* can regulate endogenous levels of *hsa-miR-206-3p* and *hsa_circ_0008726* through direct binding of the miRs on responding MREs, we transfected miR-mimic/inhibitor of *hsa-miR-381-3p*, an endogenous miR that can be downregulated by MDI-GSH conjugate exposure, into THP-1 macrophages. Given that *hsa_circ_0008726* does not contain MRE sites to *hsa-miR-381-3p*, transfection of either miR-mimic-381-3p or miR-inhibitor-381-3p had no impact on endogenous *hsa_circ_0008726* levels (Figure 3C,D). This further indicates that *hsa_circ_0008726* binds to *hsa-miR-206-3p* via the MRE site and can be potentially regulated by endogenous *hsa-miR-206-3p* in macrophages.

### 3.4. Circular RNA hsa_circ_0008726 Regulates Endogenous hsa-miR-206-3p Expression in Differentiated/Enhanced THP-1 Macrophages

**Figure 4:**
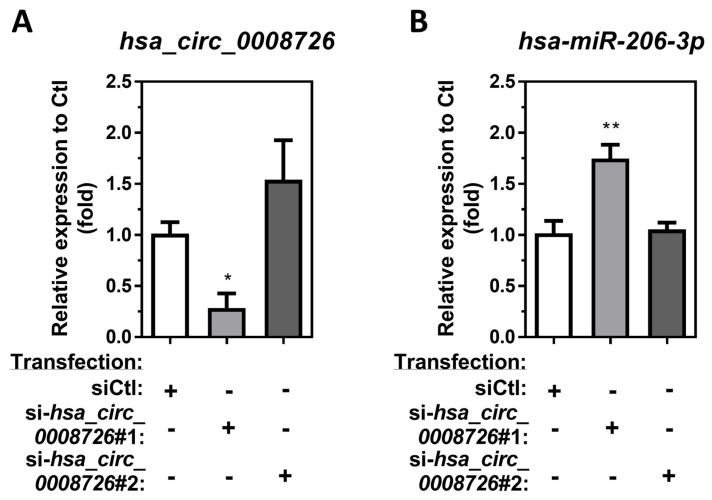
**Transfection of *hsa_circ_0008726* siRNA knocks down endogenous *hsa_circ_0008726* levels and upregulates *hsa-miR-206-3p* in differentiated/enhanced THP-1 macrophages.** Differentiated/enhanced THP-1 macrophages were transfected with 25 nM of either si-*hsa_circ_0008726*#1, si-*hsa_circ_0008726*#2 siRNA, or nontargeting siRNA control (siCtl). After 24 h, the endogenous levels of (**A**) *hsa_circ_0008726* and (**B**) *hsa-miR-206-3p* were measured by RT-qPCR (N = 3; bars, SEM). (* *p* < 0.05, ** *p* < 0.01).

To investigate the role of *hsa_circ_0008726* in regulation of *hsa-miR-206-3p*, we designed two different siRNAs for *hsa_circ_0008726* and performed a loss-of-function experiment to knockdown endogenous *hsa_circ_0008726* in differentiated/enhanced THP-1 macrophages. Differentiated/enhanced THP-1 macrophages were transfected with either si-*hsa_circ_0008726*#1 or si-*hsa_circ_0008726*#2, which were designed to target two different sites in *hsa_circ_0008726*. Transfection of si-*hsa_circ_0008726*#1 significantly inhibits the endogenous expression of *hsa_circ_0008726* levels by 3.51-fold, whereas si-*hsa_circ_0008726*#2 failed to inhibit endogenous levels of *hsa_circ_0008726* (Figure 4A). Consistent with the finding that *hsa_circ_0008726* sponges/targets endogenous *hsa-miR-206-3p* in macrophages (Figure 3), transfection of si-*hsa_circ_0008726*#1 significantly upregulated the endogenous expression of *hsa-miR-206-3p* levels by 1.75-fold, whereas si-*hsa_circ_0008726*#2 transfection failed to induce endogenous *hsa-miR-206-3p* (Figure 4B). These results suggest that endogenous *hsa_circ_0008726* can regulate endogenous *hsa-miR-206-3p* levels in macrophages.

### 3.5. Circular RNA hsa_circ_0008726 Regulates Endogenous KLF4 and M2 Macrophage-Associated Markers CD206, TGM2, CCL17, CCL22, and CCL24 Transcript Levels

**Figure 5:**
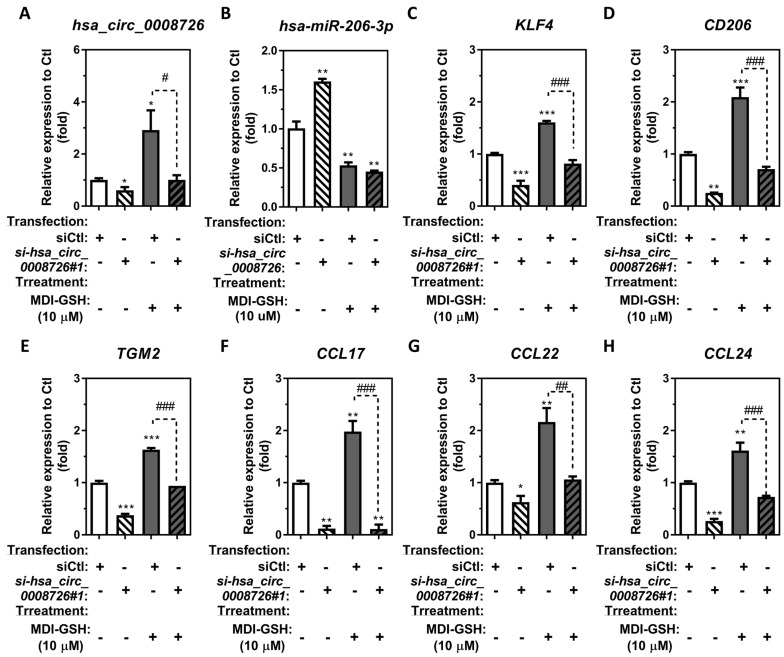
**CircRNA *hsa_circ_0008726* as a downstream effector to MDI-GSH conjugate exposure for regulating *hsa-miR-206-3p/KLF4* and KLF4-mediated M2 macrophage-associated markers and chemokines in macrophages.** Differentiated/enhanced THP-1 macrophages were transfected with 25 nM of either si-*hsa_circ_0008726*#1 or nontargeting siRNA control (siCtl) for 24 h, followed by treatment either with or without 10 µM MDI-GSH conjugate for 24 h. Total RNA was isolated from macrophages with indicated treatments/transfections by the *mirVana^™^* miR isolation kit, reverse transcribed, and subjected to SYBR green-based or TaqMan stem-loop miR RT-qPCR. The endogenous levels of (**A**) *hsa_circ_0008726* and (**B**) *hsa-miR-206-3p* as well as the M2 macrophage-associated transcription factor (**C**) *KLF4*, markers (**D**) *CD206*, (**E**) *TGM2*, (**F**) *CCL17*, (**G**) *CCL22*, and (**H**) *CCL24* mRNA levels were determined in total RNA isolated from macrophages as indicated treatments (N = 3; bars, SEM). (* *p* < 0.05, ** *p* < 0.01, *** *p* < 0.001 when compared to vehicle-treated macrophages with transfection of or nontargeting siRNA control (siCtl); ^#^ *p* < 0.05, ^##^ *p* < 0.01, ^###^ *p* < 0.001, when compared to macrophages treated with 10 µM MDI-GSH conjugate as well as with transfection of indicated either si-*hsa_circ_0008726*#1 or nontargeting siRNA control (siCtl)).

Our previous report showed that *in vivo*/*in vitro* MDI/MDI-GSH conjugate exposure upregulates transcription factor *Klf4*/KLF4 and M2 macrophage-associated markers and chemokines, including *Ccl17*/CCL17, *Ccl22*/CCL22, and *Ccl24*/CCL24, through KLF4-mediated activation in BALCs/macrophages [20]. Other reports have shown that *KLF4* transcripts can be regulated by endogenous *hsa-miR-206-3p* in many different cell types [58,59]. Recently, our laboratory reported that MDI-GSH exposure downregulates endogenous *hsa-miR-206-3p*, which directly targets the *KLF4* transcript in macrophages, inducing KLF4 and KLF4-mediated M2 macrophage-associated markers and chemokines *CD206*, *TGM2*, *CCL17*, *CCL22*, and *CCL24* transcription [22]. To study the role of *hsa_circ_0008726* in regulation of MDI-GSH conjugate-mediated induction of M2 macrophage-associated gene transcription in macrophages, we performed a loss-of-function experiment using the si-*hsa_circ_0008726*#1 siRNA to knockdown endogenous *hsa_circ_0008726* in macrophages. Transfection of si-*hsa_circ_0008726*#1 siRNA to the macrophages downregulated the endogenous *hsa_circ_0008726*, *KLF4*, *CD206*, *TGM2*, *CCL17*, *CCL22*, and *CCL24* mRNAs by 1.65-, 2.45-, 3.93-, 2.66-, 8.42-, 1.60-, and 3.80-fold, respectively (Figure 5A,C–H), whereas it upregulated endogenous *hsa-miR-206-3p* by 1.61-fold (Figure 5B) when compared to control-treated, nontargeting siCtl transfected macrophages.

Consistent with previous findings that exposure to MDI-GSH conjugate upregulates endogenous KLF4 transcription factor, M2 macrophage-associated marker and chemokine mRNA transcripts in THP-1 macrophages [20], as well as the endogenous *hsa_circ_0008726* as shown in Figure 1, independent treatment of 10 µM MDI-GSH conjugates with nontargeting siRNA control (siCtl) transfected THP-1 macrophages upregulated endogenous *hsa_circ_0008726*, *KLF4*, *CD206*, *TGM2*, *CCL17*, *CCL22*, and *CCL24* mRNAs by 2.92-, 1.61-, 2.09-, 1.63-, 1.98-, 2.16-, and 1.62-fold, respectively (Figure 5A,C–H), whereas it downregulated endogenous *hsa-miR-206-3p* by 1.88-fold (Figure 5B) compared to control-treated nontargeting siCtl transfected THP-1 macrophages. In addition, MDI-GSH conjugate treatments did not induce endogenous levels of *hsa_circ_0008726*, *KLF4*, *CD206*, *TGM2*, *CCL17*, *CCL22*, and *CCL24* mRNAs in si-*hsa_circ_0008726*#1 siRNA transfected THP-1 macrophages (Figure 5A,C–H). This suggests that *hsa_circ_0008726* may play an important role in regulating the expression of M2 macrophage-associated transcription factor KLF4, markers *CD206* and *TGM2*, as well as chemokines *CCL17*, *CCL22*, and *CCL24* following macrophage exposure to MDI-GSH conjugate.

### 3.6. Overexpression of hsa_circ_0008726 Induces Endogenous KLF4 as Well as M2 Macrophage-Associated Markers and Chemokine Transcript Expression

**Figure 6:**
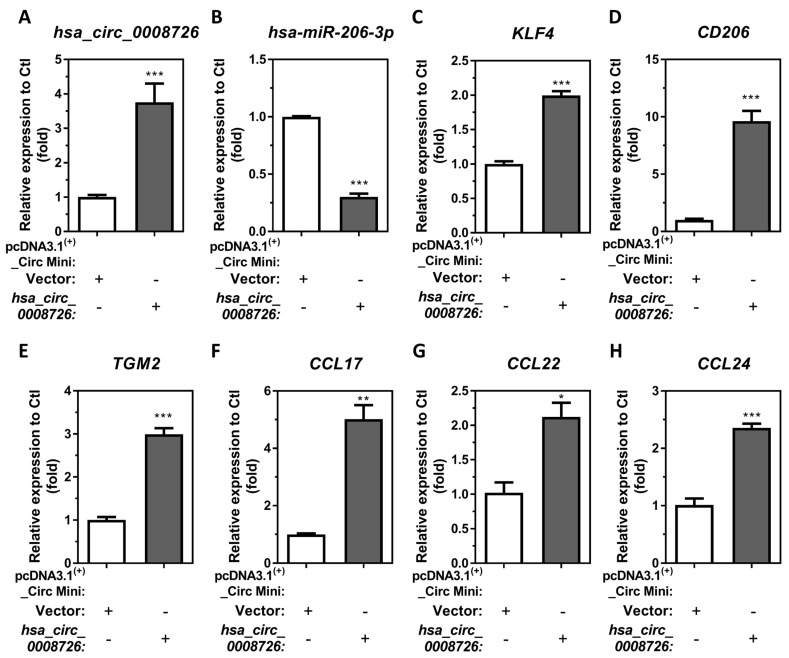
**Circular RNA *hsa_circ_0008726* overexpression increases M2 macrophage associate markers and chemokines in differentiated/enhanced THP-1 macrophages.** Differentiated/enhanced THP-1 macrophages were transfected with 2.5 µg of either pcDNA3.1^(+)^_Circ_Mini-*hsa_circ_0008726* or pcDNA3.1^(+)^_Circ_Mini vector plasmids for 48 h. Total RNA was isolated from plasmids transfected THP-1 macrophages by the *mirVana^™^* miR isolation kit, reverse transcribed, and subjected to SYBR green or TaqMan RT-qPCR. The transgene of (**A**) *hsa_circ_0008726* and (**B**) *hsa-miR-206-3p* as well as the endogenous M2 macrophage-associated markers (**C**) *KLF4*, (**D**) *CD206*, (**E**) *TGM2*, (**F**) *CCL17*, (**G**) *CCL22*, and (**H**) *CCL24* mRNA levels were determined by RT-qPCR (N = 3; bars, SEM). (* *p* < 0.05, ** *p* < 0.01, *** *p* < 0.001).

To investigate whether *hsa_circ_0008726* can mediate M2 macrophage-associated marker and chemokine transcript expression through *hsa-miR-206-3p*-regulated KLF4, we characterized the expression of M2 macrophage-associated transcription factor, markers, and chemokines *KLF4*, *CD206*, *TGM2*, *CCL17*, *CCL22*, and CCL24 using an *in vitro* *hsa_circ_0008726* overexpression model in THP-1 macrophages. Compared to vector-transfected differentiated/enhanced THP-1 macrophages, transfection of *hsa_circ_0008726* overexpression plasmids for 48 h significantly induced *hsa_circ_0008726* levels by 3.76-fold (Figure 6A), as well as endogenous *KLF4*, *CD206*, and *TGM2* mRNAs by 1.99-, 9.64-, and 2.99-fold, respectively (Figure 6C–E), whereas *hsa_circ_0008726* overexpression plasmid transfection reduced endogenous *hsa-miR-206-3p* by 3.30-fold in macrophages (Figure 6B). Furthermore, *hsa_circ_0008726* overexpression upregulated the endogenous chemokines *CCL17*, *CCL22*, and *CCL24* mRNAs by 5.02-, 2.12-, and 2.36-fold, respectively (Figure 6F–H). These results indicate that induction of *hsa_circ_0008726* following MDI-GSH conjugate exposure downregulates endogenous *hsa-miR-206-3p* while inducing *KLF4*, *CD206*, *TGM2*, *CCL17*, *CCL22*, and *CCL24* transcription and expression in human macrophages.

### 3.7. Circular RNA hsa_circ_0008726 Induces Naïve T-Cell and Eosinophil Chemoattraction by Upregulation of Chemokine CCL17, CCL22, and CCL24 in Macrophages

**Figure 7:**
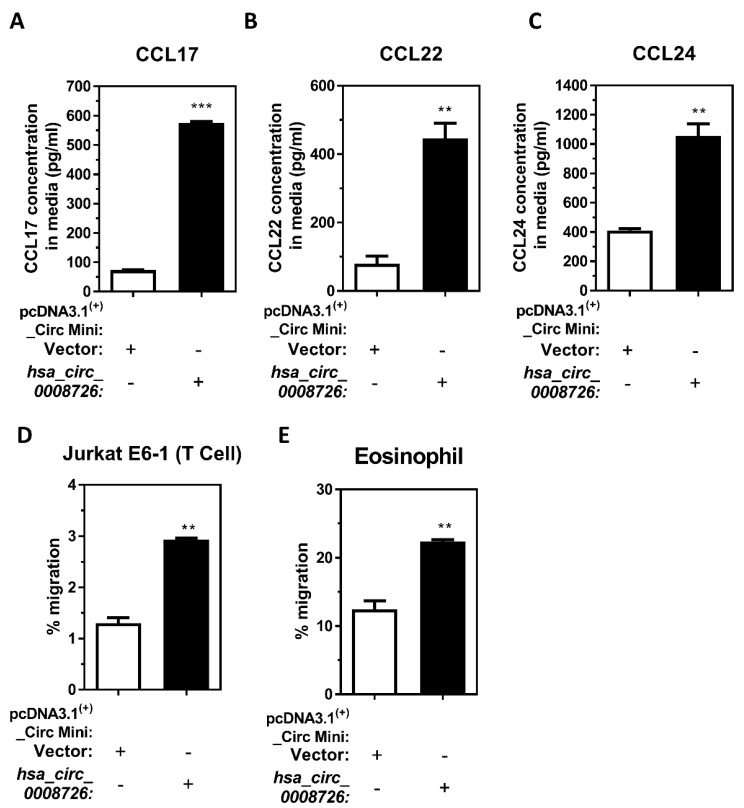
**CircRNA *hsa_circ_0008726* plays an important role for the secretion of chemokines CCL17, CCL22, and CCL24 and regulates T-cell and eosinophil chemotaxis/migration in macrophages.** Cell-free conditioned media were obtained from THP-1 macrophages transfected with either the *hsa_circ_0008726* overexpression plasmid or the empty vector for 48 h. The secreted protein levels of (**A**) CCL17, (**B**) CCL22, and (**C**) CCL24 in conditioned media from either *hsa_circ_0008726* overexpressed THP-1 macrophages or empty vector transfected THP-1 macrophages were determined by ELISA according to the manufacturer’s instructions. The isolated conditioned media were used as chemoattractants to attract (**D**) Jurkat T-cell clone E6-1 or differentiated (**E**) HL-60 C_15 eosinophils. T-cell and eosinophil migration responding to the conditioned media was measured after 6 h. Percent of cells migrated towards the bottom chamber are shown (** *p* < 0.01, *** *p* < 0.001).

M2 macrophages can secrete mediators and chemokines to attract T-cells, eosinophils, and other immune cell types [60]. In addition to T-cells, previous reports have established that CCL24 can attract eosinophils [61]. Our previous reports demonstrate that MDI-GSH conjugate-exposed macrophages attract naïve T-cells and eosinophils [20,48]. Furthermore, MDI-GSH conjugate exposure-mediated induction of M2 macrophage-associated chemokines CCL17, CCL22, and CCL24 expression may be regulated by KLF4 in macrophages. MDI-GSH conjugate exposure upregulates *hsa_circ_0008726*, *KLF4*, as well as *CCL17*, *CCL22*, and *CCL24* transcripts (Figure 5A,C,F–H) and these chemokines may contribute to T-cell, eosinophil, and other immune cell chemoattraction. We hypothesize that endogenous *hsa_circ_0008726* is involved in regulation of T-cell and/or eosinophil chemotaxis following MDI-GSH exposure in macrophages by secreting CCL17, CCL22, and CCL24. We first examined secreted CCL17, CCL22, and CCL24 protein levels in conditioned media collected from *hsa_circ_0008726* overexpression plasmid transfected differentiated/enhanced macrophages. Overexpression of *hsa_circ_0008726* induced chemokine CCL17, CCL22, and CCL24 protein levels by 7.83-, 5.63-, and 2.52-fold, respectively, when compared to vector-transfected THP-1 macrophages (Figure 7A–C). In addition, we performed chemotaxis/migration assays using conditioned media collected from *hsa_circ_0008726* overexpression plasmid transfected THP-1 macrophages. When compared to the conditioned media collected from vector-transfected differentiated/enhanced THP-1 macrophages, media collected from *hsa_circ_0008726* overexpressed macrophages induced naïve T-cell (Jurkat_E6-1 cells) chemotaxis/migration by 2.26-fold (Figure 7D), whereas it induced eosinophil (butyric acid differentiated HL-60_C15 cells) chemotaxis/migration by 1.79-fold (Figure 7E), indicating that MDI-GSH-induced *hsa_circ_0008726* plays a role in regulation of macrophage-mediated T-cell and eosinophil chemoattraction/migration.

## 4. Discussion

**Figure 8:**
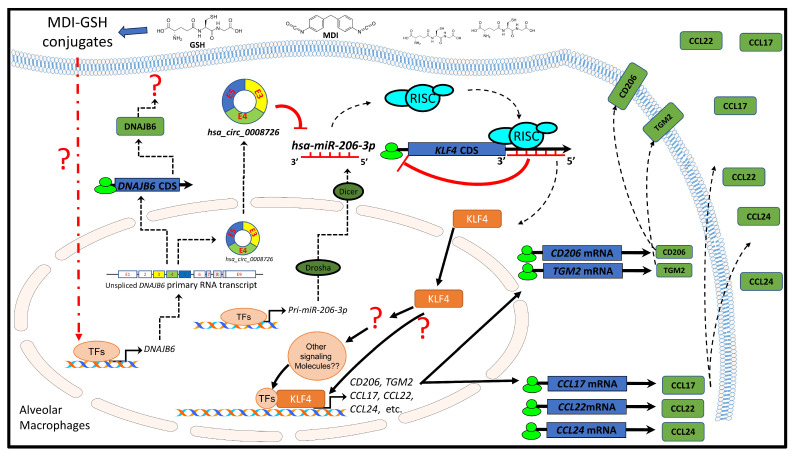
**Proposed mechanisms by which MDI-GSH conjugate exposure induces M2 macrophage-associated markers and chemokine *CCL17*, *CCL22*, and *CCL24* via *hsa_circ_0008726/hsa-miR-206-3p*-regulated KLF4 activation in macrophages.** MDI: 4,4′-methylene diphenyl diisocyanate; TFs: transcription factors; CDS: coding sequences; KLF4: Krüppel-like factor 4. Note: Some illustrated schematics were obtained from *motifolio* templates (Motifolio Inc., Ellicott City, MD, USA).

Our previous reports demonstrate that macrophages exposed to MDI/MDI-GSH downregulate endogenous *hsa-miR-206-3p* and *hsa-miR-381-3p* levels [34,35,48]; additionally, the exposure upregulates endogenous KLF4 transcription factor-mediated M2 macrophage-associated markers and chemokines *in vivo* and *in vitro*, where KLF4 may play an important role as downstream regulator/effector for MDI exposure-mediated induction of M2 macrophage-associated markers and chemokines in macrophages [20]. However, the detailed molecular mechanism(s) that result in the downregulation of *hsa-miR-206-3p/hsa-miR-381-3p* after MDI-GSH exposure is currently unknown. In the current study, we focus on the potential molecular mechanism involved in downregulating *hsa-miR-206-3p*, and we have identified a circular RNA-regulated mechanism that may be involved in the downregulation of *hsa-miR-206-3p* after MDI-GSH conjugate exposure in macrophages. Endogenous *hsa-miR-206-3p* was downregulated in an *in vitro* MDI-GSH conjugate exposure human THP-1 macrophage model as previously reported [34,48]. Furthermore, MDI-GSH conjugate exposure upregulates endogenous circular RNA *hsa_circ_0008726* and its host gene *DNAJB6* transcript in the macrophages (Figure 1 and Figure 2). The MDI-GSH exposure-induced endogenous *hsa_circ_0008726* contains the *hsa-miR-206-3p* MRE site, allowing *hsa_circ_0008726* to sponge/bind endogenous *hsa-miR-206-3p*, causing downregulation/degradation of endogenous *hsa-miR-206-3p* after MDI-GSH conjugate exposure. Additionally, downregulation of endogenous *hsa-miR-206-3p* results in the upregulation of KLF4 transcription factor expression. Induction of KLF4 and KLF4-mediated activation of downstream gene transcription upregulates expression of M2 macrophage-associated markers and chemokines, including *CD206*, *TGM2*, *CCL17*, *CCL22*, and *CCL24* transcripts. (Figure 5 and Figure 6). Using an *in vitro* THP-1 macrophage culture model, we identified that *hsa_circ_0008726* sponges/binds endogenous *hsa-miR-206-3p*, resulting in downregulation/degradation of *hsa-miR-206-3p*, which targets the *KLF4* mRNA transcript, suppresses KLF4 translation, and decreases *KLF4* transcripts in macrophages (Figure 8). Although *hsa_circ_0008726* has been reported to target *hsa-miR-206-3p* in other cell types, including esophageal squamous cell carcinoma and fibroblast cells [42,43], to our knowledge, the current report is the first to identify and verify that *hsa_circ_0008726* is expressed in macrophages and can regulate the expression of endogenous *hsa-miR-206-3p* and *hsa-miR-206-3p*-mediated posttranscriptional regulation of endogenous gene function in macrophages.

Macrophages exposed to MDI-GSH conjugate induced endogenous *hsa_circ_0008726* through upregulation of *hsa_circ_0008726* host gene *DNAJB6* primary transcript production (Figure 2); however, the mechanism by which MDI-GSH conjugate exposure induces *DNAJB6* is currently unknown. The induction of *DNAJB6* transcripts may be cellular stress responses within the macrophages in response to the MDI/MDI-GSH conjugate exposure. The human DNAJB6 protein is a member of the DNAJ protein family, which functions as one of the two major classes of molecular chaperones involved in different cellular processes, such as protein folding regulation, oligomeric protein complex assembly, and toxic protein aggregation prevention [62]. It has been implicated in a variety of molecular pathways associated with cellular stress responses in different cell types, including cellular responses to stimuli, heat stresses, and viral infections [63,64,65,66,67]. DNAJB6 is involved in the pathogenesis of many different types of cancers [68,69,70,71]. In colon cancer cells, DNAJB6 promotes cell adhesion, migration, and invasion through stabilizing the urokinase plasminogen activator receptor (uPAR), as well as activating many signaling transduction proteins through phosphorylation, including FAK, ERK1/2, and AKT [71]. In the colon macrophages, the uPAR promotes phagocytosis and regulates macrophage polarization toward the M2-phenotype [72]. Although the function of DNAJB6 in macrophages has been shown to be a cellular stress response to regulate the different stages of viral replication and infection [73], the role of DNAJB6 in macrophage polarization has never been investigated. We speculate that the induction of *DNAJB6* transcription in macrophages exposed to MDI/MDI-GSH conjugate may facilitate macrophage uPAR stabilization and therefore promote M2 macrophage polarization and phagocytosis. Further investigation into the roles of DNAJB6 and macrophage polarization is needed. Furthermore, the detailed molecular mechanism(s) by which MDI/MDI-GSH conjugate induces *DNAJB6* transcription should be investigated to understand the role of DNAJB6 in MDI-OA pathogenesis.

Dysfunction of alveolar macrophages has been shown to play an important role in asthma pathogenesis and treatment [16]. The dysfunction of molecular gene regulation and gene expression homeostasis affects the role of alveolar macrophages, such as macrophage polarization and production/secretion of macrophage mediators. Among the gene regulators that contribute to alveolar macrophage dysfunction in asthma, circRNA has been suggested as a major player in asthma progression and pathogenesis [74]. Using an OVA-induced asthma mouse model, Shang et al. revealed that murine circRNA *mmu_circ_0001359* was significantly decreased in OVA-treated mice, and murine *mmu_circ_0001359* was involved in protective effects that prevent asthma development in the mice [75]. Murine *mmu_circ_0001359* contributes to M2 macrophage polarization through sponging/targeting *mmu-miR-183-5p* with MRE sites on *mmu_circ_0001359* and induces *FoxO1*, a *mmu-miR-183-5p* target. Induction of FoxO1 attenuated airway remodeling by reducing M1 macrophage activation-induced inflammatory cytokine expression and the progression of pulmonary fibrosis [75]. Similarly, Gong et al. demonstrated that *hsa_circ_0001326* prevented THP-1 macrophages from undergoing M2 polarization, instead promoting the macrophages toward M1 polarization by directly regulating the *hsa-miR-136-5p/USP4* axis, which decreases the expression and secretion of pro-inflammatory factors TNF-α and IL-6 and instead promotes the expression of anti-inflammatory factor IL-10 [76]. These studies suggest endogenous circRNAs in macrophages may participate in asthma pathogenesis by modulating M2 macrophage polarization via targeting the miRs involved. Given that MDI exposure in the form of MDI-GSH conjugate promotes M2 macrophage-associated markers and chemokines that represent M2 macrophage polarization, whether MDI-GSH conjugate exposure may affect endogenous human homologues of murine *mmu_circ_0001359*, *hsa_circ_0001326*, or other M2 macrophage-promoting circRNA(s) will be of interest in future studies.

The role of endogenous circRNAs in macrophages that contribute to M2 macrophage polarization and activation has been recently demonstrated in human and murine asthma models. Liang et al. demonstrated that *hsa_circ_0014208* (*hsa_circ_S100A11*) is highly expressed in monocytes and remarkably upregulated in children with asthma [77]. The circRNA *hsa_circ_0014208* promotes the translation of its host gene *S100A11*, inducing S100A11 protein expression in the macrophages. S100A11 proteins contribute to M2 macrophage polarization through SP3-mediated induction of STAT6, which promotes M2 macrophage polarization and activation [77]. Using a cockroach allergen-induced murine asthma model, Liang et al. demonstrated that the S100A11 protein exacerbates lung inflammation by increasing the production and secretion of Th2 chemokines CCL17 and CCL22 in lung macrophages, further inducing inflammatory pulmonary injury in allergic asthma [77]. Similar to Liang’s observation that *hsa_circ_0014208* promotes translation of its host gene *S100A11* as well as production and secretion of Th2 chemokines CCL17 and CCL22, our current report demonstrates that *hsa_circ_0008726* promotes the expression and secretion of M2 macrophage-associated chemokines CCL17, CCL22, and CCL24 as well as the chemotactic ability of naïve T-cells and eosinophils through induction of KLF4 transcription factor expression via targeting *hsa-miR-206-3p* (Figure 6 and Figure 7). Although the function of the *hsa_circ_0008726* host gene *DNAJB6* in regulation of M2 macrophage-associated chemokines CCL17, CCL22, and CCL24 is currently unknown, future studies must characterize the role of DNAJB6 in macrophages. Furthermore, whether MDI/MDI-GSH conjugate exposure can induce expression of *hsa_circ_0014208* given its demonstrated role in allergic pathogenesis should be investigated.

Strengths of the current *in vitro* THP-1 macrophage MDI-GSH conjugate exposure model include the ability to perform gain- or loss-of-function experiments with miR mimics/inhibitors and circRNA *hsa_circ_0008726* overexpression plasmid transfections, as well as the siRNA transfection experiments to knockdown endogenous *hsa_circ_0008726* with identical genetics from a single cell type to investigate the underlying circRNA-mediated molecular regulatory mechanisms involved in observed MDI-GSH conjugate-mediated effects in macrophages. However, one inherent limitation of the model is that it leaves uncertain whether or not the identified *hsa_circ_0008726*-mediated induction of KLF4 via downregulation of endogenous *hsa-miR-206-3p* and the *hsa_circ_0008726/hsa-miR-206-3p*/KLF4 regulatory axis-mediated induction of M2 macrophage-associated markers and chemokines mechanism reflects similar lung immune responses that may participate in the early steps of asthma pathogenesis in occupational MDI-OA. Furthermore, the current study is based on the identification of candidate *hsa-miR-206-3p* sponging/targeting circRNA from the published literature. This experimental design omits other potential *hsa-miR-206-3p* targeting circRNAs that may be regulated by MDI-GSH conjugate exposure in macrophages. Future studies will be needed to better elucidate potential connections of diisocyanate exposure with circRNA/miR targeting gene regulatory mechanisms in real-world MDI workers.

## 5. Conclusions

In conclusion, this report implicates *hsa_circ_0008726* as an important regulator of *hsa-miR-206-3p*, *KLF4* transcripts, and KLF4-mediated signaling, ultimately targeting M2 macrophage-associated markers and chemokine transcription in macrophages after MDI-GSH conjugate exposure. MDI-GSH conjugate exposure in an *in vitro* human THP-1 macrophage cell culture model results in the induction of *hsa_circ_0008726* and downregulation of endogenous *hsa-miR-206-3p*. This *hsa_circ_0008726/hsa-miR-206-3p/KLF4* regulatory axis may contribute to upregulation of M2 macrophage-associated markers and chemokines, including *CD206*, *TGM2*, *CCL17*, *CCL22*, and *CCL24* transcripts in macrophages following MDI/MDI-GSH exposure. The circRNA *hsa_circ_0008726* may play an important role in regulation of MDI-OA pathogenesis through macrophage-mediated chemokine expression and induction of immune cell chemotaxis to the lung microenvironment.

## Data Availability

All data required to evaluate the conclusions of this paper are included in the main text and the Results Section.

## Supplementary Materials

The following supporting information can be downloaded at: https://www.mdpi.com/article/10.3390/cells13201725/s1, Table S1: Candidate *hsa-miR-206-3p* binding circRNA divergent primer sequences; Figure S1: Screenshot from CircBank showing no predicted binding between *hsa-miR-381-3p* and *hsa_circ_0008726*.

## References

[1] Allport D.C., Gilbert D.S., Outterside S.M. (2003). MDI and TDI: A Safety, Health and the Environment: A Source Book and Practical Guide.

[2] Statista (2024). Demand for Methylene Diphenyl Diisocyanate Worldwide from 2011 to 2023. https://www.statista.com/statistics/750809/mdi-demand-worldwide/.

[3] NIOSH (1994). Letter from NIOSH to Distinctive Designs International Inc. with a Study Report. https://www.cdc.gov/niosh/hhe/reports/pdfs/1991-0386-2427.pdf.

[4] Lofgren D.J., Walley T.L., Peters P.M., Weis M.L. (2003). MDI Exposure for Spray-On Truck Bed Lining. Appl. Occup. Environ. Hyg..

[5] Redlich C.A., Karol M.H. (2002). Diisocyanate asthma: Clinical aspects and immunopathogenesis. Int. Immunopharmacol..

[6] Engfeldt M., Isaksson M., Zimerson E., Bruze M. (2013). Several cases of work-related allergic contact dermatitis caused by isocyanates at a company manufacturing heat exchangers. Contact Dermat..

[7] Jan R.L., Chen S.H., Chang H.Y., Yeh H.J., Shieh C.C., Wang J.Y. (2008). Asthma-like syndrome in school children after accidental exposure to xylene and methylene diphenyl diisocyanate. J. Microbiol. Immunol. Infect..

[8] Wisnewski A.V., Cooney R., Hodgson M., Giese K., Liu J., Redlich C.A. (2022). Severe asthma and death in a worker using methylene diphenyl diisocyanate MDI asthma death. Am. J. Ind. Med..

[9] Cantin A.M., North S.L., Hubbard R.C., Crystal R.G. (1987). Normal alveolar epithelial lining fluid contains high levels of glutathione. J. Appl. Physiol. (1985).

[10] Day B.W., Jin R., Basalyga D.M., Kramarik J.A., Karol M.H. (1997). Formation, solvolysis, and transcarbamoylation reactions of bis(S-glutathionyl) adducts of 2,4- and 2,6-diisocyanatotoluene. Chem. Res. Toxicol..

[11] Reisser M., Schmidt B.F., Brown W.E. (2002). Synthesis, characterization, and solvolysis of mono- and bis-S-(glutathionyl) adducts of methylene-bis-(phenylisocyanate) (MDI). Chem. Res. Toxicol..

[12] Wisnewski A.V., Liu J., Redlich C.A. (2013). Connecting glutathione with immune responses to occupational methylene diphenyl diisocyanate exposure. Chem. Biol. Interact..

[13] Barnes P.J. (2008). Immunology of asthma and chronic obstructive pulmonary disease. Nat. Rev. Immunol..

[14] Holgate S.T. (2008). Pathogenesis of asthma. Clin. Exp. Allergy.

[15] Boonpiyathad T., Sozener Z.C., Satitsuksanoa P., Akdis C.A. (2019). Immunologic mechanisms in asthma. Semin. Immunol..

[16] Fricker M., Gibson P.G. (2017). Macrophage dysfunction in the pathogenesis and treatment of asthma. Eur. Respir. J..

[17] Girodet P.O., Nguyen D., Mancini J.D., Hundal M., Zhou X., Israel E., Cernadas M. (2016). Alternative Macrophage Activation Is Increased in Asthma. Am. J. Respir. Cell Mol. Biol..

[18] Wisnewski A.V., Liu J., Redlich C.A. (2020). Analysis of Lung Gene Expression Reveals a Role for Cl(-) Channels in Diisocyanate-induced Airway Eosinophilia in a Mouse Model of Asthma Pathology. Am. J. Respir. Cell Mol. Biol..

[19] Wisnewski A.V., Liu J., Colangelo C.M. (2015). Glutathione reaction products with a chemical allergen, methylene-diphenyl diisocyanate, stimulate alternative macrophage activation and eosinophilic airway inflammation. Chem. Res. Toxicol..

[20] Lin C.C., Law B.F., Hettick J.M. (2023). 4,4′-Methylene diphenyl diisocyanate exposure induces expression of alternatively activated macrophage-associated markers and chemokines partially through Kruppel-like factor 4 mediated signaling in macrophages. Xenobiotica.

[21] Park C.S., Shen Y., Lewis A., Lacorazza H.D. (2016). Role of the reprogramming factor KLF4 in blood formation. J. Leukoc. Biol..

[22] Lin C.C., Law B.F., Hettick J.M. (2024). MicroRNA-mediated Kruppel-like factor 4 upregulation induces alternatively activated macrophage-associated marker and chemokine transcription in 4,4′-methylene diphenyl diisocyanate exposed macrophages. Xenobiotica.

[23] Peng F., Gong W., Li S., Yin B., Zhao C., Liu W., Chen X., Luo C., Huang Q., Chen T. (2021). *circRNA_010383* Acts as a Sponge for *miR-135a*, and Its Downregulated Expression Contributes to Renal Fibrosis in Diabetic Nephropathy. Diabetes.

[24] Ding T., Zhu Y., Jin H., Zhang P., Guo J., Zheng J. (2021). Circular RNA *circ_0057558* Controls Prostate Cancer Cell Proliferation Through Regulating *miR-206*/USP33/c-Myc Axis. Front. Cell Dev. Biol..

[25] Zhu H., Zhu S., Shang X., Meng X., Jing S., Yu L., Deng Y. (2021). Exhausting *circ_0136474* and Restoring *miR-766-3p* Attenuate Chondrocyte Oxidative Injury in IL-1beta-Induced Osteoarthritis Progression Through Regulating DNMT3A. Front. Genet..

[26] Zeng H., Gao H., Zhang M., Wang J., Gu Y., Wang Y., Zhang H., Liu P., Zhang X., Zhao L. (2021). Atractylon Treatment Attenuates Pulmonary Fibrosis via Regulation of the mmu_circ_0000981/miR-211-5p/TGFBR2 Axis in an Ovalbumin-Induced Asthma Mouse Model. Inflammation.

[27] Huang Z., Cao Y., Zhou M., Qi X., Fu B., Mou Y., Wu G., Xie J., Zhao J., Xiong W. (2019). *Hsa_circ_0005519* increases IL-13/IL-6 by regulating *hsa-let-7a-5p* in CD4(+) T cells to affect asthma. Clin. Exp. Allergy.

[28] Salmena L., Poliseno L., Tay Y., Kats L., Pandolfi P.P. (2011). A ceRNA hypothesis: The Rosetta Stone of a hidden RNA language?. Cell.

[29] Zhang Y., Zhang Y., Li X., Zhang M., Lv K. (2017). Microarray analysis of circular RNA expression patterns in polarized macrophages. Int. J. Mol. Med..

[30] Zhang J., Cheng F., Rong G., Tang Z., Gui B. (2021). Circular RNA *hsa_circ_0005567* overexpression promotes M2 type macrophage polarization through *miR-492*/SOCS2 axis to inhibit osteoarthritis progression. Bioengineered.

[31] Wang Y., Gao R., Li J., Tang S., Li S., Tong Q., Li S. (2021). Downregulation of *hsa_circ_0074854* Suppresses the Migration and Invasion in Hepatocellular Carcinoma via Interacting with HuR and via Suppressing Exosomes-Mediated Macrophage M2 Polarization. Int. J. Nanomed..

[32] Zhang C., Han X., Yang L., Fu J., Sun C., Huang S., Xiao W., Gao Y., Liang Q., Wang X. (2020). Circular RNA *circPPM1F* modulates M1 macrophage activation and pancreatic islet inflammation in type 1 diabetes mellitus. Theranostics.

[33] Song H., Yang Y., Sun Y., Wei G., Zheng H., Chen Y., Cai D., Li C., Ma Y., Lin Z. (2022). Circular RNA *Cdyl* promotes abdominal aortic aneurysm formation by inducing M1 macrophage polarization and M1-type inflammation. Mol. Ther..

[34] Lin C.C., Law B.F., Hettick J.M. (2020). Acute 4,4′-Methylene Diphenyl Diisocyanate Exposure-Mediated Downregulation of *miR-206-3p* and *miR-381-3p* Activates Inducible Nitric Oxide Synthase Transcription by Targeting Calcineurin/NFAT Signaling in Macrophages. Toxicol. Sci..

[35] Lin C.C., Law B.F., Siegel P.D., Hettick J.M. (2019). Circulating *miRs-183-5p, -206-3p* and *-381-3p* may serve as novel biomarkers for 4,4′-methylene diphenyl diisocyanate exposure. Biomarkers.

[36] Patop I.L., Wust S., Kadener S. (2019). Past, present, and future of circRNAs. EMBO J..

[37] Zhang B., Huo S., Cen X., Pan X., Huang X., Zhao Z. (2020). *circAKT3* positively regulates osteogenic differentiation of human dental pulp stromal cells via miR-206/CX43 axis. Stem Cell Res. Ther..

[38] Wang Y., Guo T., Liu Q., Xie X. (2020). CircRAD18 Accelerates the Progression of Acute Myeloid Leukemia by Modulation of *miR-206/*PRKACB Axis. Cancer Manag. Res..

[39] Li H., Xia Z., Liu L., Pan G., Ding J., Liu J., Kang J., Li J., Jiang D., Liu W. (2021). Astragalus IV Undermines Multi-Drug Resistance and Glycolysis of MDA-MB-231/ADR Cell Line by Depressing *hsa_circ_0001982-miR-206/miR-613* Axis. Cancer Manag. Res..

[40] Mei X., Cui X.B., Li Y., Chen S.Y. (2021). *CircSOD2*: A Novel Regulator for Smooth Muscle Proliferation and Neointima Formation. Arterioscler. Thromb. Vasc. Biol..

[41] Wang X., Bai M. (2021). CircTM7SF3 contributes to oxidized low-density lipoprotein-induced apoptosis, inflammation and oxidative stress through targeting *miR-206*/ASPH axis in atherosclerosis cell model in vitro. BMC Cardiovasc. Disord..

[42] Zhang Y., Fang S., Wang J., Chen S., Xuan R. (2022). Hsa_circ_0008726 regulates the proliferation, migration, and invasion of trophoblast cells in preeclampsia through modulating the miR-1290-LHX6 signaling pathway. J. Clin. Lab. Anal..

[43] Han T., Shi M., Chen G., Hao J. (2023). *Circ_0008726* promotes malignant progression of ESCC cells through *miR-206*/HOXA13 pathway. Gen. Thorac. Cardiovasc. Surg..

[44] Zheng X., Ma Y.F., Zhang X.R., Li Y., Zhao H.H., Han S.G. (2020). *Circ_0056618* promoted cell proliferation, migration and angiogenesis through sponging with *miR-206* and upregulating CXCR4 and VEGF-A in colorectal cancer. Eur. Rev. Med. Pharmacol. Sci..

[45] Li H., Yao G., Feng B., Lu X., Fan Y. (2018). *Circ_0056618* and CXCR4 act as competing endogenous in gastric cancer by regulating miR-206. J. Cell Biochem..

[46] Feng W., Han S. (2022). lncRNA *ADAMTS9-AS1/circFN1* Competitively Binds to miR-206 to Elevate the Expression of ACTB, Thus Inducing Hypertrophic Cardiomyopathy. Oxid. Med. Cell Longev..

[47] Wang M., Gao Y., Liu J. (2019). Silencing circZFR inhibits the proliferation, migration and invasion of human renal carcinoma cells by regulating miR-206. Onco Targets Ther..

[48] Lin C.C., Law B.F., Hettick J.M. (2021). MicroRNA-mediated calcineurin signaling activation induces CCL2, CCL3, CCL5, IL8, and chemotactic activities in 4,4′-methylene diphenyl diisocyanate exposed macrophages. Xenobiotica.

[49] Maess M.B., Wittig B., Cignarella A., Lorkowski S. (2014). Reduced PMA enhances the responsiveness of transfected THP-1 macrophages to polarizing stimuli. J. Immunol. Methods.

[50] Baxter E.W., Graham A.E., Re N.A., Carr I.M., Robinson J.I., Mackie S.L., Morgan A.W. (2020). Standardized protocols for differentiation of THP-1 cells to macrophages with distinct M(IFNgamma+LPS), M(IL-4) and M(IL-10) phenotypes. J. Immunol. Methods.

[51] Fischkoff S.A. (1988). Graded increase in probability of eosinophilic differentiation of HL-60 promyelocytic leukemia cells induced by culture under alkaline conditions. Leuk. Res..

[52] Tiffany H.L., Li F., Rosenberg H.F. (1995). Hyperglycosylation of eosinophil ribonucleases in a promyelocytic leukemia cell line and in differentiated peripheral blood progenitor cells. J. Leukoc. Biol..

[53] Badewa A.P., Hudson C.E., Heiman A.S. (2002). Regulatory effects of eotaxin, eotaxin-2, and eotaxin-3 on eosinophil degranulation and superoxide anion generation. Exp. Biol. Med. (Maywood).

[54] Liang D., Wilusz J.E. (2014). Short intronic repeat sequences facilitate circular RNA production. Genes. Dev..

[55] Lin C.C., Liu L.Z., Addison J.B., Wonderlin W.F., Ivanov A.V., Ruppert J.M. (2011). A KLF4-miRNA-206 autoregulatory feedback loop can promote or inhibit protein translation depending upon cell context. Mol. Cell Biol..

[56] Dudekula D.B., Panda A.C., Grammatikakis I., De S., Abdelmohsen K., Gorospe M. (2016). CircInteractome: A web tool for exploring circular RNAs and their interacting proteins and microRNAs. RNA Biol..

[57] Liu M., Wang Q., Shen J., Yang B.B., Ding X. (2019). Circbank: A comprehensive database for circRNA with standard nomenclature. RNA Biol..

[58] Parasramka M.A., Dashwood W.M., Wang R., Saeed H.H., Williams D.E., Ho E., Dashwood R.H. (2012). A role for low-abundance miRNAs in colon cancer: The miR-206/Kruppel-like factor 4 (KLF4) axis. Clin. Epigenetics.

[59] Tang X., Tian X., Zhang Y., Wu W., Tian J., Rui K., Tong J., Lu L., Xu H., Wang S. (2013). Correlation between the frequency of Th17 cell and the expression of microRNA-206 in patients with dermatomyositis. Clin. Dev. Immunol..

[60] Mantovani A., Sica A., Sozzani S., Allavena P., Vecchi A., Locati M. (2004). The chemokine system in diverse forms of macrophage activation and polarization. Trends Immunol..

[61] White J.R., Imburgia C., Dul E., Appelbaum E., O’Donnell K., O’Shannessy D.J., Brawner M., Fornwald J., Adamou J., Elshourbagy N.A. (1997). Cloning and functional characterization of a novel human CC chemokine that binds to the CCR3 receptor and activates human eosinophils. J. Leukoc. Biol..

[62] Hageman J., Rujano M.A., van Waarde M.A.W.H., Kakkar V., Dirks R.P., Govorukhina N., Oosterveld-Hut H.M.J., Lubsen N.H., Kampinga H.H. (2010). A DNAJB Chaperone Subfamily with HDAC-Dependent Activities Suppresses Toxic Protein Aggregation. Mol. Cell.

[63] Kumar M., Mitra D. (2005). Heat Shock Protein 40 Is Necessary for Human Immunodeficiency Virus-1 Nef-mediated Enhancement of Viral Gene Expression and Replication*. J. Biol. Chem..

[64] Cheng X., Belshan M., Ratner L. (2008). Hsp40 facilitates nuclear import of the human immunodeficiency virus type 2 Vpx-mediated preintegration complex. J. Virol..

[65] Kumar M., Rawat P., Khan S.Z., Dhamija N., Chaudhary P., Ravi D.S., Mitra D. (2011). Reciprocal regulation of human immunodeficiency virus-1 gene expression and replication by heat shock proteins 40 and 70. J. Mol. Biol..

[66] Pei Y., Fu W., Yang E., Shen A., Chen Y.C., Gong H., Chen J., Huang J., Xiao G., Liu F. (2012). A Hsp40 Chaperone Protein Interacts with and Modulates the Cellular Distribution of the Primase Protein of Human Cytomegalovirus. PLoS Pathog..

[67] Fan C.Y., Lee S., Cyr D.M. (2003). Mechanisms for regulation of Hsp70 function by Hsp40. Cell Stress. Chaperones.

[68] Sterrenberg J.N., Blatch G.L., Edkins A.L. (2011). Human DNAJ in cancer and stem cells. Cancer Lett..

[69] Mitra A., Menezes M.E., Shevde L.A., Samant R.S. (2010). DNAJB6 Induces Degradation of β-Catenin and Causes Partial Reversal of Mesenchymal Phenotype*. J. Biol. Chem..

[70] Mitra A., Fillmore R.A., Metge B.J., Rajesh M., Xi Y., King J., Ju J., Pannell L., Shevde L.A., Samant R.S. (2008). Large isoform of MRJ (DNAJB6) reduces malignant activity of breast cancer. Breast Cancer Res..

[71] Lin Y., Peng N., Zhuang H., Zhang D., Wang Y., Hua Z.C. (2014). Heat shock proteins HSP70 and MRJ cooperatively regulate cell adhesion and migration through urokinase receptor. BMC Cancer.

[72] Genua M., D’Alessio S., Cibella J., Gandelli A., Sala E., Correale C., Spinelli A., Arena V., Malesci A., Rutella S. (2015). The urokinase plasminogen activator receptor (uPAR) controls macrophage phagocytosis in intestinal inflammation. Gut.

[73] Ko S.H., Huang L.M., Tarn W.Y. (2019). The Host Heat Shock Protein MRJ/DNAJB6 Modulates Virus Infection. Front. Microbiol..

[74] Liu X., Ali M.K., Dua K., Mao Y., Liu J. (2023). Circular RNAs: Emerging players in asthma and COPD. Front. Cell Dev. Biol..

[75] Shang Y., Sun Y., Xu J., Ge X., Hu Z., Xiao J., Ning Y., Dong Y., Bai C. (2020). Exosomes from *mmu_circ_0001359*-Modified ADSCs Attenuate Airway Remodeling by Enhancing FoxO1 Signaling-Mediated M2-like Macrophage Activation. Mol. Ther. Nucleic Acids.

[76] Gong B., Zheng Y., Li J., Lei H., Liu K., Tang J., Peng Y. (2022). Luteolin activates M2 macrophages and suppresses M1 macrophages by upregulation of hsa_circ_0001326 in THP-1 derived macrophages. Bioengineered.

[77] Liang Q., Fu J., Wang X., Liu L., Xiao W., Gao Y., Yang L., Yu H., Xie X., Tu Z. (2023). *circS100A11* enhances M2a macrophage activation and lung inflammation in children with asthma. Allergy.

