# Supplementary material for: Circular RNA hsa_circ_0008726 Targets the hsa-miR-206-3p/KLF4 Axis to Modulate 4,4′-Methylene Diphenyl Diisocyanate-Glutathione Conjugate-Induced Chemokine Transcription in Macrophages
Source: Cells. 2024 Oct 18;13(20):1725. doi: 10.3390/cells13201725 (PMC11505732; doi:10.3390/cells13201725)
Supplement: Supplementary file 1 [file cells-13-01725-s001.zip › cells-3221644-supplementary.pdf]

Supplemental Table 1. Candidate *hsa-miR-206-3p* binding circRNA divergent primer sequences.

Candidate circRNA	Forward	Reverse	Host Gene	Host gene Accession #
hsa_circ_0000199	ATCATTGCTTTCAGGGCTCT	CACCCGCTCTCTCGACAAAT	*AKT3*	NM_181690
hsa_circ_0001264	CAGCTCATTAAAAGGCACCA	GGAAGAAGCAGGAGATTTGG	*RAD18*	NM_020165
hsa_circ_0001982	CAATCCAGCTGTTCCCTCAG	TGGTGCATCAGAAGGAATCTC	*RNF111*	NM_017610
hsa_circ_0004662	GTGTGGGAGCACGCTTACTA	CGTTAGGGCTGAGGTTTGTC	*SOD2*	NM_001024465
hsa_circ_0007428	GCAGGAGAGGGTAGTTGTGC	TCCCCAAGTACCAAGTGCAT	*SH3BP4*	NM_014521
hsa_circ_0008726	GGTGGAAGGGACCCATTTTC	TGCCTCCGCTACTTGCTTG	*DNAJB6*	NM_058246
hsa_circ_0056618	GAACCCACCCCACCTCTAC	CTTCCCCGGGATAAACAAACC	*SPOPL*	NM_001001664
hsa_circ_0057558	AGTCACTGCAGGCATGTTC	TGCAACAAGGAATGTAAGA	*SLC39A10*	NM_001127257
hsa_circ_0058141	GCCCTCAATTCATTCCAATG	TGAGGCTCTCTTCTTCATCA	*FN1*	NM_212482
hsa_circ_0072088	ACGCATTCTTCGAGACCTCT	TGCCTGTAACTCCTCTTCAGT	*ZFR*	NM_016107

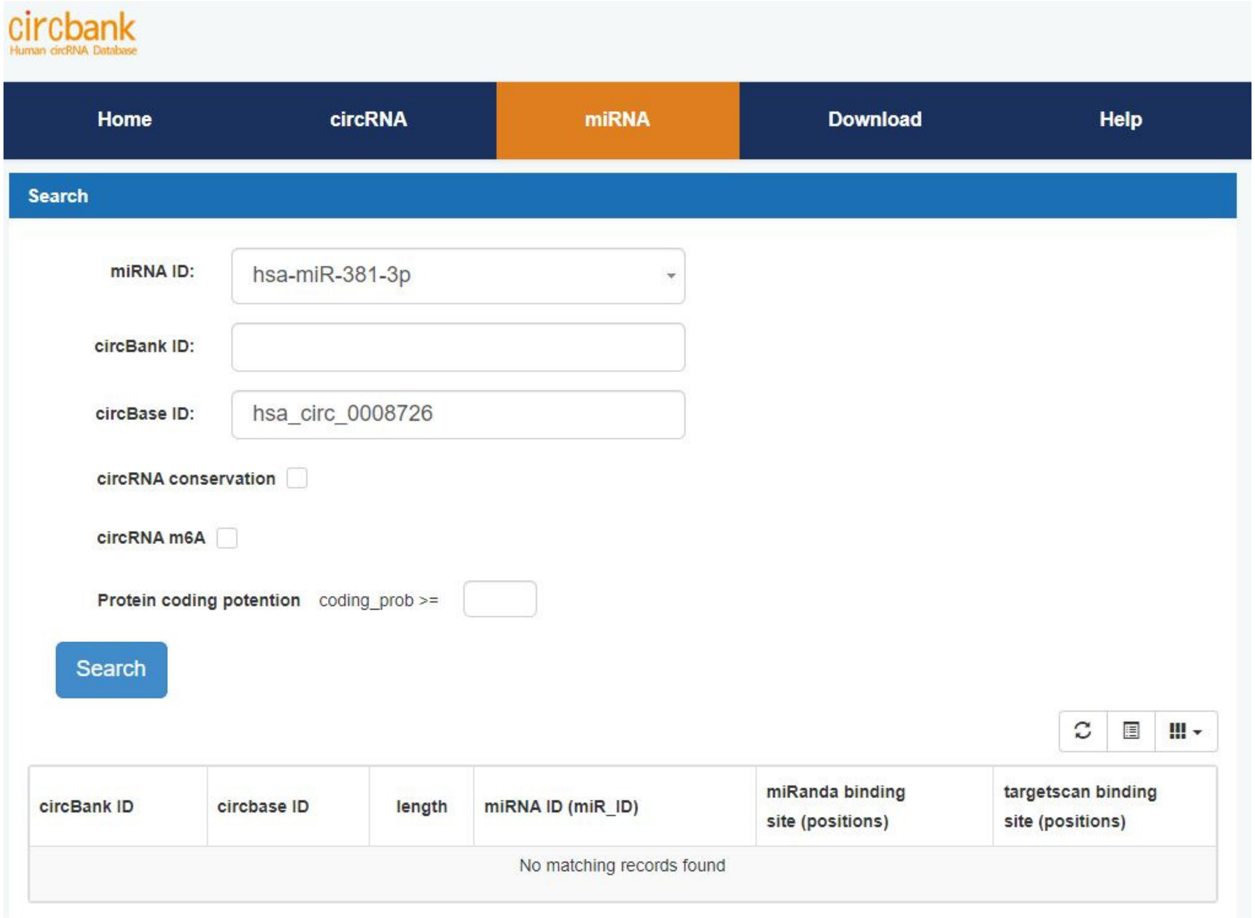

Figure S1. Screenshot from CircBank (http://www.circbank.cn/searchMiRNA.html; accessed July 8th, 2024), showing no predicted binding between *hsa-miR-381-3p* and *hsa_circ_0008726*.
